# Comparative Evaluation of Combined Denoising and Resolution Enhancement Algorithms for Intravital Two-Photon Imaging of Organs

**DOI:** 10.3390/bios15090616

**Published:** 2025-09-17

**Authors:** Saeed Bohlooli Darian, Woo June Choi, Jeongmin Oh, Jun Ki Kim

**Affiliations:** 1 Department of Biomedical Engineering, College of Medicine, University of Ulsan, Seoul 05505, Republic of Korea; 2 School of Electrical and Electronics Engineering, Chung-Ang University, Seoul 06974, Republic of Korea; cecc78@cau.ac.kr; 3 Department of Intelligent Semiconductor Engineering, Chung-Ang University, Seoul 06974, Republic of Korea; 4 Convergence Medicine, Asan Institute for Life Sciences, Asan Medical Center, Seoul 05505, Republic of Korea; mini1kr@mail.ulsan.ac.kr

**Keywords:** computational enhancement, denoising, eSRRF, mitochondria, super-resolution imaging, two-photon intravital microscopy

## Abstract

Intravital two-photon microscopy enables deep-tissue imaging of subcellular structures in live animals, but its original spatial resolution and image quality are limited by scattering, motion, and low signal-to-noise ratios. To address these challenges, we used a combination of tissue stabilization, denoising methods, and motion correction, together with resolution enhancement algorithms, including enhanced Super-Resolution Radial Fluctuations (eSRRF) and deconvolution, to acquire high-fidelity time-lapse images of internal organs. We applied this imaging pipeline to image genetically labeled mitochondria in vivo, in Dendra2 mice. Our results demonstrate that the eSRRF-combined method, compared to other evaluated algorithms, significantly shows improved spatial resolution and mitochondrial structure visualization, while each method exhibiting distinct strengths in terms of noise tolerance, edge preservation, and computational efficiency. These findings provide a practical framework for selecting enhancement strategies in intravital imaging studies targeting dynamic subcellular processes.

## 1. Introduction

By enabling the direct observation of biological dynamics in living systems, intravital imaging [1,2] has significantly advanced the biological sciences. Among optical imaging modalities [3,4,5,6,7], two-photon microscopy [8,9] gained prominence due to its potential to attain deep tissue penetration with low photobleaching and phototoxicity, rendering it a preferred tool for live animal investigations. Microendoscopic probe- and gradient-index (GRIN)-based imaging modalities [10,11] have further increased the reach of two-photon microscopy to deeper or inaccessible tissues. Despite these advantages, conventional two-photon microscopy remains hampered by its diffraction-limited resolution [12] and is vulnerable to biological artifacts such as tissue motion [13] and autofluorescent signals [14]. To overcome these limitations, numerous studies have explored enhancing image quality and resolution by introducing modifications to the microscope’s optical setup [15,16]. Although sub-diffraction calibration using nano beads provides a more accurate measure of absolute resolution, such standards are not feasible in live intravital settings. Therefore, our study focused on evaluating relative resolution improvements under physiological conditions, rather than asserting nanoscale resolving power.

Mitochondria [17,18] have been a key target for intravital imaging. These submicron organelles play critical roles in cellular metabolism [19], calcium regulation [20], and apoptotic signaling [21]. Mitochondria constantly change shape through fission and fusion [22,23] processes, closely linked to the cell’s physiological state and disease. In metabolically active tissues like the liver, observing mitochondria in real time can reveal important information about cellular health. However, imaging mitochondria in living organisms is difficult due to their small size (usually less than 5 μm) [24], light scattering by tissues, and motion artifacts.

As emphasized, motion of tissues is a significant challenge, particularly in high-resolution imaging studies. The primary sources of motion are respiration, blood flow, and cardiac pulsation. Minimal movements may blur details or give false impressions of mitochondrial transition. To address this issue, a stabilization technique that integrates physical limitations with imaging windows [25] and computational motion correction are critical in maintaining spatial accuracy throughout live imaging. This method minimizes motion artifacts, maintains a stable FOV, and enables high-resolution imaging throughout long imaging sessions.

To overcome the resolution limits of traditional imaging methods, super-resolution techniques [26,27] such as STED, SIM, and PALM have been developed to circumvent this limitation through computational or optical innovations. However, these hardware-intensive methods are not compatible with live animal imaging due to light toxicity, slow frame rates, or limited depth penetration [28].

By contrast, computational super-resolution techniques that utilize temporal fluorescence fluctuations offer a software-based alternative. Of these, enhanced Super-Resolution Radial Fluctuations (eSRRF) [29,30] is recognized as a robust algorithm that is capable of reconstructing high-resolution images from standard time-lapse data without requiring further setup alterations. Deconvolution [31] is another common technique used to enhance clarity of images, computationally unwarping optical distortions to increase visual detail. To further improve these algorithms’ capabilities in resolution-enhancement, additional preprocessing steps, such as denoising, are used to eliminate background noises. With respect to typical procedures used in eliminating noises, we used Gaussian and median filtering, as well as a trained model called Noise2Noise (N2N) [32], to achieve a noise-free image. To quantitatively determine our methods’ efficiency, rolling Fourier Ring Correlation (rFRC) [33] analysis serves as a trustworthy metric used to test image local resolution and give an evaluation into the effectiveness of used techniques.

Achieving optimal results in intravital imaging requires the selection of the most suitable image processing algorithm. Although all these techniques show results of optimizing images, comparatively analyzing these techniques under challenging settings, like in applications involving hepatic tissue or other highly vascular tissues, is relevant and should be investigated further in more demanding in vivo settings. Therefore, there is a need to evaluate the enhancing capabilities of image enhancement algorithms in vivo settings, to find the best suiting ones.

In this study, using genetically labeled mitochondria mice, we demonstrate and assess intravital imaging of diverse organs under physiological conditions through an in vivo imaging framework that combines two-photon microscopy, tissue stabilization, denoising algorithms, and sophisticated computational resolution improvement algorithms. We contrasted a combination of these algorithms for improvement in resolution, structural fidelity, and computational processing speed in live animal imaging. This is a robust technique applicable to high-resolution subcellular imaging in vivo and adds a practical standard following which suitable enhancement procedures have to be chosen for complex biological systems.

## 2. Materials and Methods

### 2.1. Animal Preparation

All animal experiments were approved by the Institutional Animal Care and Use Committee (IACUC) of Asan Medical Center. Dendra2 transgenic mice (6–8 weeks old, 25–30 g, strain#: 018397, Jackson Laboratory, Bar Harbor, ME, USA) were used for all imaging experiments. Mice were housed in cages of five at 23 °C with access to food and water, under a 12 h dark/light cycle. Prior to imaging, animals were anesthetized with a mixture of Zoletil (Virbac, Carros, France), Rompun (Elanco, Greenfield, IN, USA), and DPBS (Thermo Fisher, Waltham, MA, USA) (6:4:90, 10 mL/kg), intubated, and ventilated using a MiniVent 845 small animal ventilator (Harvard Apparatus, Holliston, MA, USA). Additional anesthesia was administered promptly upon detection of any animal movement to ensure complete immobilization and prevent motion artifacts during imaging. Body temperature was maintained at 37 °C using the microscope heated stage during all procedures.

### 2.2. Intravital Imaging Procedure

Following anesthesia, the fur was shaved to provide a clear target for imaging. First, the abdominal skin was chosen for imaging due to its easy accessibility but also because it poses a challenge due to significant motion artifacts created by heartbeat-induced vibrations. Next, to directly visualize hepatocytes, a midline incision was made in the abdomen to expose a lobe of liver, which was carefully lifted and placed against the stabilizer window. For cardiac imaging, the chest was opened and stabilized with a retractor to gain direct access to the heart. For skeletal muscle imaging, the thigh skin was removed to reveal the quadriceps. The kidney and brain were accessed through dorsal and cranial incisions exposing the skull or flank, keeping the organs in their natural positions while minimizing bleeding and stress. Extreme care was taken to avoid injury to both the target organs and surrounding tissues.

After imaging, skin incisions were thoroughly sutured and disinfected to ensure proper wound closure. The mice were placed on a heated recovery pad to maintain body temperature during recovery. Once their condition was stable, they were returned to their cages and monitored for future imaging sessions.

In vivo imaging was carried out using a commercial two-photon microscopy system (IVM-MS, IVIM Technology, Daejeon, Republic of Korea) equipped with a Ti–Sapphire laser (Coherent Inc., Santa Clara, CA, USA) set to 920 nm for excitation. Fine structural details were captured by detecting mitochondrial fluorescence signal with a high-magnification objective lens (100×, NA 1.45, Olympus, Tokyo, Japan) and broadband detectors (185–760 nm) to ensure efficient signal collection. Imaging was performed at 1024 × 1024 pixels and 15 frames per second with a pixel size of 105 nm for high-resolution analysis. Optical sectioning for 3D reconstruction was performed with Z-step imaging with 1 µm increments.

Tissue stabilization was achieved by applying gentle negative pressure to the tissue surface using a custom designed 3D-printed holder to facilitate intravital imaging, as previously described [25]. The portable window is compatible with various objective lenses, enabling stable and flexible intravital imaging across different experimental setups. The system effectively immobilized the tissue, minimizing motion artifacts without causing physical damage or physiological stress by carefully selecting the minimum pressure value required for optimal stabilization. A coverslip separates the tissue and objective lens, enabling the use of immersion media for refractive index matching and the best optical performance.

### 2.3. Image Analysis

The obtained image stacks were processed using Fiji 1.54 software package [34]. Fluorescence signals were then retrieved to illustrate the regions of interest. Background noises were then eliminated to improve the signal-to-noise ratio and prepare the raw data optimally for further analysis via applying common algorithms: (1) Gaussian filtering, (2) Median filtering, (3) a denoising model. Three various sigma settings (0.5, 1, 2) for Gaussian and Median filtering were used to examine and compare the results. The denoising model was executed using a trained self-supervised denoising model via the N2N plugin in Fiji. To eliminate motion artifacts that still lingered, pixel shifts in the time series of images were determined and a registration algorithm used to reduce these movements. To enhance resolution and image clarity, preprocessed image stacks were then processed further via eSRRF and deconvolution methods through the NanoJ [35] and DeconvolutionLab [36] plugins, respectively. For eSRRF, parameter optimization was performed via sweeping sensitivity and ring radius settings to achieve optimal reconstruction through quality and resolution (QnR) map [37] computation. Deconvolution was then conducted following PSF (point spread function) calculation to ensure proper restoration of structural details.

Mitochondrial morphology was analyzed using the Mitochondrial Network Analysis workflow (MiNA) [38] in Fiji. The method calculated significant parameters including average branch length and network size. Additionally, other morphological features such as circularity and mitochondrial coverage area were assessed through particle analysis tool in Fiji.

To evaluate the effectiveness of this approach, we compared the resolution of standard and enhanced images and computed their respective rFRC maps using a 1/7 threshold via the PANELJ plugin [33], to validate the resolution improvement.

### 2.4. Alcoholic Liver Disease Model

To illustrate an example of our suggested workflow, we explored alcohol’s impact on hepatocyte mitochondria [39,40]. Mice were given ethanol (EtOH; 70%, 6 mg/kg, Daejung Reagent Chemicals, Shiheung Si, Republic of Korea) orally before imaging. The baseline imaging occurred immediately after administration to give us the control dataset. Imaging took place 2–3 h after treatment to assess mitochondrial morphology peak changes caused by alcohol. Quantification of morphological parameters like mitochondrial length and circularity were computed to evaluate prospective transition states of mitochondrial dynamics.

## 3. Results

**Figure 1:**
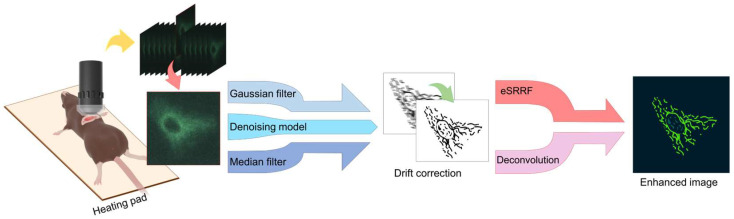
Overview of the imaging workflow combining tissue-stabilized two-photon microscopy with image processing steps, including denoising, drift correction, and resolution enhancement. Denoising was performed using Gaussian and median filtering, and a trained denoising model. The resulting images were further enhanced using either the eSRRF or deconvolution algorithm for improved visualization.

We established an intravital imaging protocol that combines a tissue stabilization method with two-photon microscope to allow visualization of subcellular structures at high resolution in vivo. The protocol has two main components: (1) a physical imaging system and (2) a sophisticated image processing pipeline. As illustrated in Figure 1, after image acquisition, the image processing pipeline initiates with filtering and background noise subtraction to remove background illumination artifacts and improve visualization. If necessary, a drift-correcting image registration algorithm is applied prior to enhance resolution via eSRRF or deconvolution to resolve single subcellular structures.

### 3.1. Quantitative Evaluation of Resolution Enhancement Techniques

In this study, we evaluated different popular image improvement techniques to find out the best method suitable for visualization of mitochondria in skeletal muscle fibers, where they are intricately arranged between myofibrils in a highly organized pattern. Gaussian and median filtering decreased background noise but also blurred out edge sharpness and fine structural information. The denoising technique, meanwhile, maintained mitochondrial borders and increased overall contrast.

**Figure 2:**
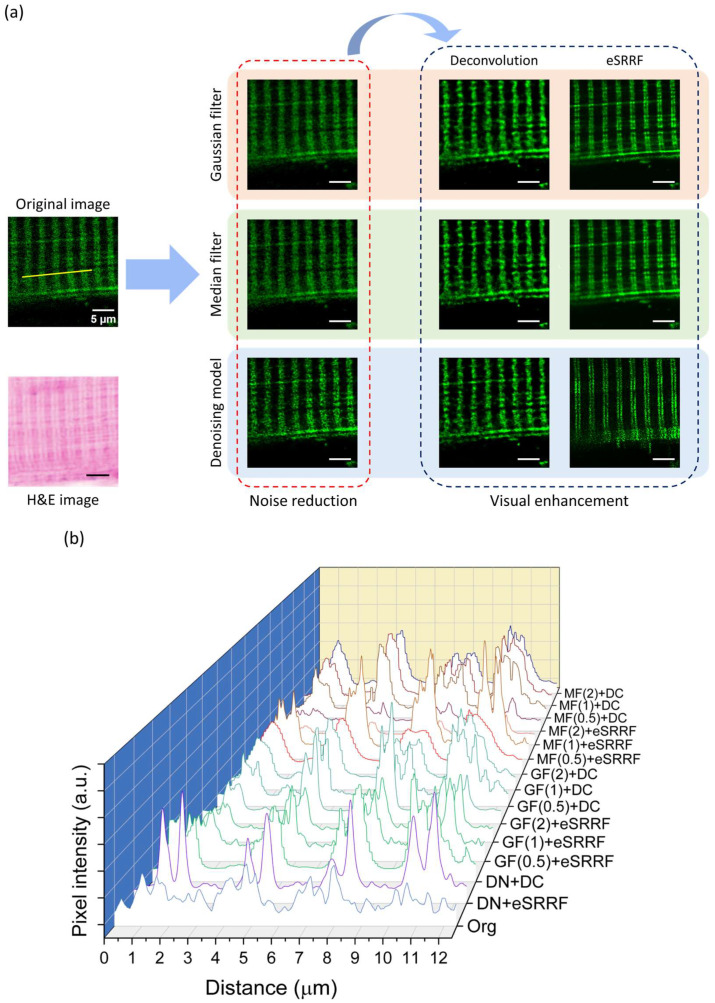
Evaluation of image enhancement methods for mitochondrial imaging. (**a**) Representative quadriceps muscle images processed with different techniques. While Gaussian and median filters reduced noise, they also blurred structural edges. Denoising model preserved detail, and combining it with eSRRF produced the clearest visualization of individual mitochondria. (**b**) Pixel intensity profiles measured along the indicated mitochondrial structures reveal variations in signal intensity and peak resolution across different denoising and enhancement approaches. For Gaussian and median filtering, sigma values of 0.5, 1, and 2 were applied, highlighting the impact of these parameters on the clarity and contrast of mitochondrial structures. (Org: Original, DN: Denoising model, DC: Deconvolution, GF: Gaussian filter, MF: Median filter). Scale bars: 5 µm.

Following noise reduction step, eSRRF application provided more quantitatively and visually superior images compared to deconvolution. Mitochondrial boundaries became distinctly defined, allowing visualization of individual mitochondria in dense regions (Figure 2a). For comparison, H&E-stained sections of the tissue were also imaged to confirm the presence of striated structures, supported by reference to additional literature sources [41,42]. Consistent with visual observation, pixel intensity along illustrated profiles indicated smaller noise and differentiated peaks in eSRRF–denoising combination-method images as opposed to other processed images (Figure 2b).

**Figure 3:**
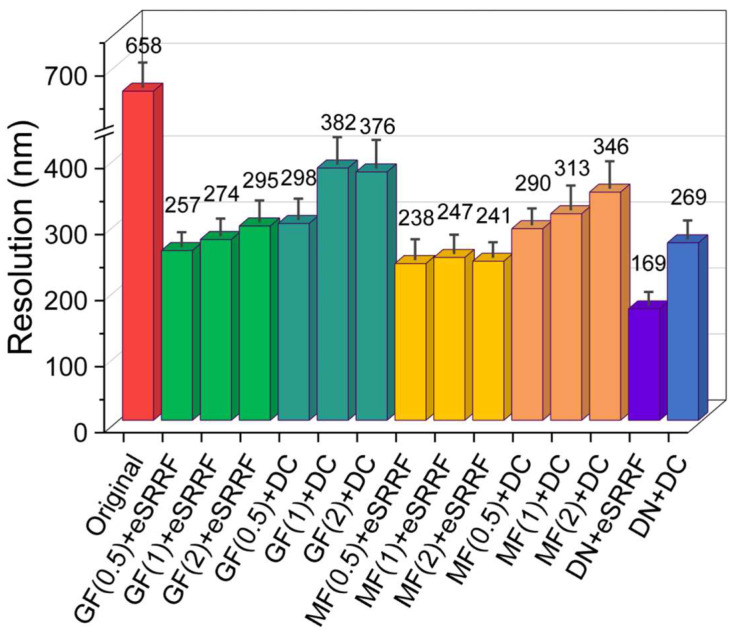
Resolution comparison of different denoising and visual enhancement methods evaluated by rFRC analysis. Images were processed using denoising model, Gaussian, and median filters with sigma values of 0.5, 1, and 2, in combination with eSRRF and deconvolution. The resulting resolution metrics highlight the performance trade-offs between conventional filters and more sophisticated enhancement techniques. Error bar: standard deviation (GF: Gaussian filter, MF: Median filter, DN: Denoising model, DC: Deconvolution).

In addition, rFRC analysis further confirmed the effectiveness of our investigations by resolution evaluation. Raw images exhibited an average local resolution of approximately 658 nm, while images processed with eSRRF combined with the denoising model achieved resolutions below 170 nm in optimized regions where there is sufficient SNR. Deconvolution improved the resolution to around 250–300 nm but often introduced ringing artifacts in some frames, which could compromise image interpretability. Although the overall resolution differences among various processing combinations were significant, the eSRRF-based approaches consistently outperformed deconvolution-based methods, demonstrating superior resolution and fewer artifacts. Moreover, eSRRF provided more stable resolution improvement across time points and different tissue regions, based on the consistency of rFRC values over sequential frames. A detailed comparison of resolution outcomes for the various processing workflows is presented in Figure 3.

### 3.2. Imaging Various Organs

**Figure 4:**
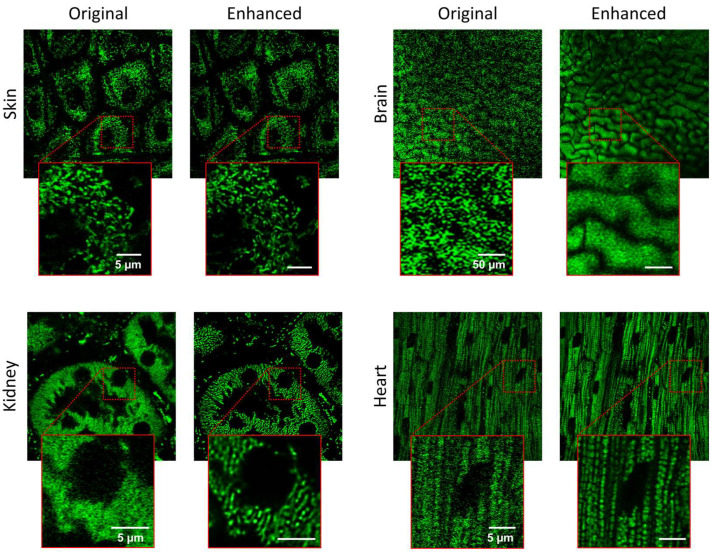
Application of the enhancement pipeline across multiple organs. Mitochondrial structures are visualized in skin, brain, kidney, and heart tissues following resolution enhancement. The distinct morphology of mitochondria in each tissue reflects organ-specific structural and anatomical characteristics, such as the densely packed, aligned fibers in cardiac muscle, the branched forms in brain tissue, and the epithelial patterns seen in skin and kidney.

Our enhancement pipeline was implemented on various tissues such as skin, kidney, brain, and heart with the same processing parameters to showcase its robustness and generalizability (Figure 4). In skin tissue, elongated and tubular mitochondria are readily resolved in the epidermal layers. In kidney, the processed images show mitochondria along the definitive tubular and glomerular arrangements that are typical of kidney anatomy. In the brain, where intense scattering and autofluorescence usually mask subtle details, the denoising step served a critical purpose in effectively eliminating background noise and bring out mitochondrial morphology. In heart tissue, despite motion artifacts due to cardiac contractions are a serious issue, our stabilization algorithm reduced motion blur significantly, enabling clear visualization of cardiac muscle fibers and corresponding mitochondria in cardiac tissue.

### 3.3. Three-Dimensional Mitochondrial Mapping of Hepatocytes

**Figure 5:**
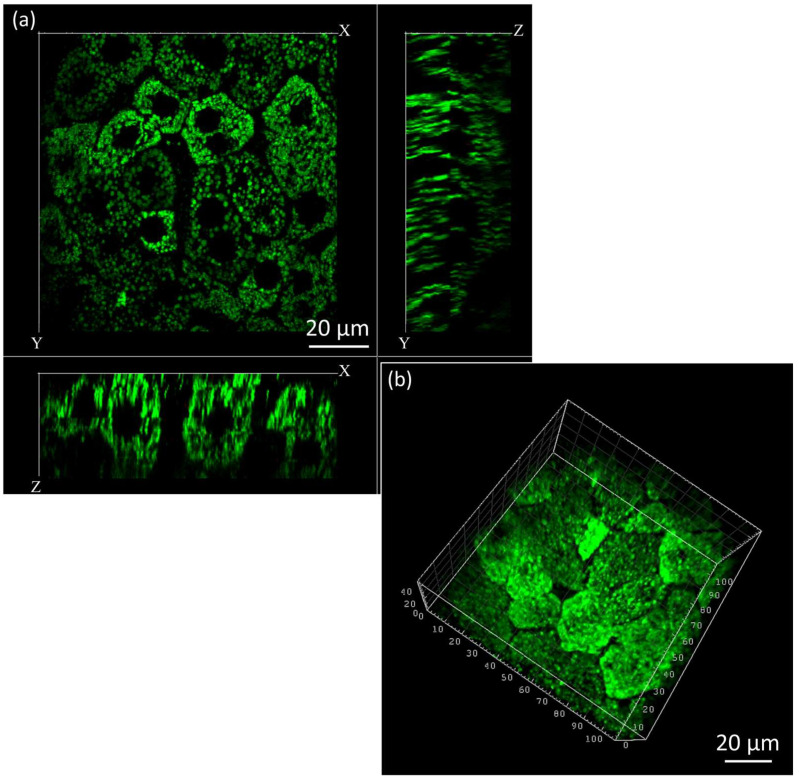
Three-dimensional reconstruction of mitochondrial networks in hepatocytes. (**a**) 50 µm depth-resolved section view showing mitochondrial distribution at 1 µm intervals. (**b**) 3D reconstruction reveals mitochondria and regional density variations, highlighting metabolic heterogeneity. Increased brightness was applied to visualize low-intensity cells, resulting in oversaturation in brighter areas.

By acquiring Z-stacks at 1 µm intervals to a depth of 50 µm, we reconstructed three-dimensional mitochondrial networks of hepatocytes (Figure 5) to demonstrate stabilization capability. The 3d-reconstructed morphologies enabled us to interrogate not only inter-mitochondrial relations but also local variation in mitochondrial densities that implicate underlying metabolic heterogeneity among hepatocytes.

Both in single-plane and 3D views, mitochondrial membrane potential differences caused fluorescence intensity changes that made a few cells brighter compared to others [43]. To visualize cells with lower signal intensities, we increased overall illumination of images, which caused a few brighter cell areas to become oversaturated as a result. This compensation was employed to view both simultaneously occurring high- and low-intensity signals throughout a given field of view so that a complete range of mitochondrial structure might be assessed.

### 3.4. Alcohol-Induced Mitochondrial Fragmentation Detection

**Figure 6:**
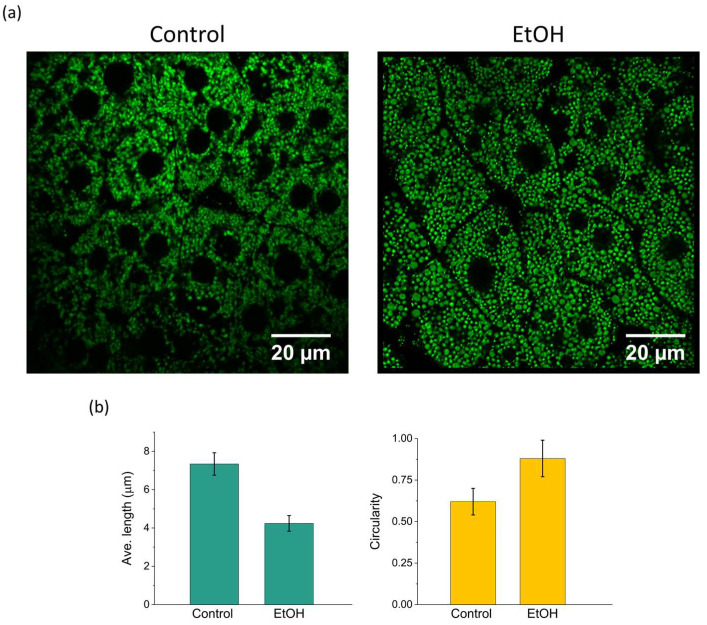
In vivo imaging of mitochondrial dynamics in an acute alcoholic liver injury model. (**a**) Representative image showing increased mitochondrial fragmentation in hepatocytes after EtOH administration. (**b**) Quantitative analysis reveals a higher fragmentation and circularity index and reduced average mitochondrial length compared to controls, indicating elevated fission activity. Error bar: standard deviation.

To show the biological significance and a practical application of our imaging pipeline, we used this method to image an acute alcoholic liver injury model in mice. Upon administration of EtOH, we detected remarkable morphological alterations of mitochondria in hepatocytes as soon as 2–3 h following treatment (Figure 6a). Relative to control hepatocytes, EtOH -treated cells had mitochondria that were clearly rounder and more fragmented, typical of a transition toward more mitochondrial fission. This fragmentation is a characteristic early sign of mitochondrial dysfunction and cellular stress in liver damage. The fragmentation was quantified by calculating the inverse of the average mitochondrial length, obtained through mitochondrial network analysis.

Quantitative morphometric analysis supported these findings by revealing a significant decrease in the mitochondrial length as well as a marked increase in circularity value, in the EtOH-treated group (Figure 6b). Together, these results demonstrate that our imaging pipeline enables the detection of subtle and dynamic changes in mitochondrial morphology under pathological stress in vivo, providing valuable insights into the early stages of liver injury progression.

## 4. Discussion

This study presents a practical and efficient workflow for enhancing subcellular resolution in intravital imaging by combining two-photon microscopy, tissue stabilization, denoising, and computer-based super-resolution algorithms. Imaging live dynamic organelles in vivo is problematic due to a demand for adequate spatial resolution, motion artifacts, scattering of light in thick tissues, and low signal-to-noise ratios. Mitochondria are particularly challenging imaging targets due to their submicron size, high density within cells, and rapid morphological dynamics such as fission and fusion. Accurately capturing these features requires both high spatial resolution and minimal motion artifacts.

The results shown here illustrate that physical stabilization and computational resolution enhancement techniques need to be incorporated in combination to get over these limitations and clearly see mitochondrial networks in different organs without losing physiological conditions. The stabilization of tissue step was critical to eliminate motion blur and provided longitudinally reliable imaging, and the denoising step significantly enhanced the quality of raw images without adding artificial texture and over-smoothing.

Amongst the popular evaluated denoising and resolution enhancement algorithms, Gaussian filtering, median filtering, and a trained deep-learning-based denoising model, with enhanced Super-Resolution Radial Fluctuations (eSRRF) and deconvolution, were selected for this investigation. These algorithms were selected because they are applicable extensively under fluctuating imaging conditions and universally outperform others in recovering spatial resolution. Specifically, Gaussian, and median filters provide a simple but effective suppression of background noises by smoothing out intensity variations, while the denoising model employs learned features of images to inhibit background noises without losing fine details of images. eSRRF excels at extracting sub-diffraction structural details by analyzing temporal fluctuations in the signal, enabling the visualization of features beyond the conventional resolution limit. Meanwhile, deconvolution sharpens images by computationally reversing the blurring introduced by the microscope’s point spread function (PSF), thereby restoring true structural information. Together, these approaches offer a powerful toolkit for enhancing in vivo imaging data, enabling clearer visualization of fine biological structures critical for this study.

The combination of the denoising model and eSRRF consistently provided the highest spatial precision while maintaining computational efficiency including the processing speed, resolution performance, and the hardware requirements, making it suitable for live imaging workflows. Its ability to extract sub-diffraction spatial features from conventional time-lapse data, without requiring phototoxic light doses or hardware modifications, represents a significant advantage for in vivo applications. However, when applying resolution enhancement algorithms, caution must be taken to avoid the introduction of image processing artifacts. Therefore, validation using previous references or alternative imaging modalities that offer higher-quality images without heavy processing should serve as a useful approach for confirmation.

The rFRC-based resolution quantification confirmed that the eSRRF-processed images had improved structural definition compared to other techniques. In visual comparisons, mitochondrial profiles processed through eSRRF revealed finer detail and higher contrast, especially when preceded by denoising and motion correction. This allowed not only static observation of subcellular morphology but also dynamic tracking of mitochondrial changes in response to physiological and pathological stimuli, as investigated previously [25].

The alcoholic liver disease model clearly showcased the application of our approach: after exposing the tissue to EtOH, we observed mitochondrial fragmentation, indicating increased fission activity and cellular stress. These dynamic changes in mitochondrial shape are essential for understanding how liver disease develops, and our imaging pipeline made it possible to directly visualize these alterations in living tissue with minimal invasiveness.

Additionally, we demonstrated the robustness of this pipeline across a variety of tissues. By applying it to liver, skeletal muscle, kidney, brain, and heart samples, we confirmed that the method adapts well to organs with different blood vessel densities, optical properties, and motion patterns. The ability to consistently enhance image quality across such diverse tissues highlights the broad applicability of our approach, which is vital for advancing translational biomedical research. Notably, our 3D reconstructions of hepatocytes using z-stacked images further show improved segmentation fidelity, clearer borders, and less background interference that this method can generate volumetric data well-suited for detailed morphometric analysis. However, despite significant advancements in lateral resolution, axial resolution remains a limiting factor and warrants further investigation. Improving resolution along the *z*-axis is essential for accurate three-dimensional visualization of subcellular structures, especially in thick or scattering tissues where optical sectioning becomes more challenging. Also, some elongation was observed along the axial direction, which may be attributed to refractive index mismatch, or to focal drift during imaging [44]. Additional optical calibration or adjustments may be necessary to correct this distortion and achieve more accurate 3D representation.

While the eSRRF algorithm demonstrated superior visual detail and ease of implementation compared to the other evaluated methods, it should be emphasized that no single resolution enhancement technique can be considered universally superior, as there are a wide range of algorithms and their performance may vary depending on imaging conditions and experimental context. Factors like residual motion, uncorrected noise, or signal loss can all interfere with enhancement algorithms, leading to distorted or misleading results. This is why thorough preprocessing steps are essential parts of any imaging workflow. Moreover, although the lateral resolution and visualization of the mitochondria are improved by the eSRRF algorithm, it does not resolve individual mitochondria in all organs. The mitochondrial morphology varies between the organs. For example, mitochondria in skeletal muscles exhibit a highly arranged pattern that aligns with the direction of the muscle fiber, smaller in size and closely packed [45]. On the other hand, the mitochondria network in the organ cells tend to have less strict arrangement, round and randomly spread across the cell [46,47]. However, as presented in the results in Figure 4, the suggested algorithm succeeded in enhancing the resolution to the point that the single mitochondria in the organ cells, such as skin and kidney cells are clearly visible.

In the end, our approach provides a scalable, non-invasive method for high-resolution imaging of fine structures in living tissues. This method is highly adaptable to a wide range of biological studies focused on subcellular structures like lysosomes and autophagosomes, metabolic disorders, and organ physiology, even in their natural unstable physiological environments. Moreover, unlike deep-learning super-resolution methods such as Deep-STORM [48,49] which depend on sparse single-molecule localization and are unsuitable for imaging dense, fast-moving structures in scattering live tissues, our integrated denoising and eSRRF approach offers a practical and reliable solution for visualizing detailed mitochondrial networks and other crowded organelles. While our current demonstration was conducted using a single imaging setup, the core components of the method are primarily based on post-processing and can be considered hardware-independent. It is fully compatible with standard two-photon microscopy systems and does not require specialized fluorophores beyond conventional fluorescent labeling. To ensure broader applicability, more validation will involve applying this workflow across diverse imaging platforms and modalities in our future studies.

## 5. Conclusions

We introduce a reliable pipeline for high-resolution intravital imaging that combines two-photon microscopy with tissue stabilization, denoising, and resolution enhancement techniques. This method enables detailed visualization of mitochondrial morphology in living tissues and was demonstrated using a standard two-photon system without hardware modifications. In this study, we applied this approach to the acute alcoholic liver injury model to observe the temporal mitochondria morphology. The comparative evaluation of common image enhancement algorithms offered practical insights based on our evaluated conditions, though we acknowledge that no single technique is universally optimal. Future studies will expand the application of this pipeline to additional disease models and imaging systems.

## Data Availability

The data presented in this study will be available on a request.

## Abbreviations

The following abbreviations are used in this manuscript:

eSRRF	Enhanced super-resolution radial fluctuations
EtOH	Ethanol
N2N	noise2noise
PSF	Point Spread Function
QnR	quality and resolution
rFRC	rolling Fourier ring correlation
